# First record of a *Sarcocystis felis*-like sarcocyst in skeletal muscle of the Eurasian lynx (*Lynx lynx*)

**DOI:** 10.1186/s13028-026-00873-6

**Published:** 2026-08-03

**Authors:** Arvid Uggla, Erik Ågren

**Affiliations:** 1 Department of Animal Biosciences, Faculty of Veterinary Medicine and Animal Science, Swedish University of Agricultural Sciences (SLU), PO Box 7023, 750 07 Uppsala, Sweden; 2 Department of Pathology and Wildlife Diseases, Swedish Veterinary Agency (SVA), 751 89 Uppsala, Sweden

**Keywords:** Felidae, Sarcocyst, Sweden, Ultrastructure

## Abstract

A *Sarcocystis* sarcocyst was found in skeletal muscle of an adult female Eurasian lynx (*Lynx lynx*) killed in 1999 in central Sweden and subjected to necropsy as part of a nationwide monitoring program. The sarcocyst was microscopic, and ultrastructurally it had a relatively thin (~1 mm) sarcocyst wall with pleomorphic villar protrusions of Type 9 according to the classification of Dubey et al. [3]. This is the first record of a *S. felis*-like sarcocyst in the Eurasian lynx (*L. lynx*).

## Background

The Eurasian lynx (*Lynx lynx*) is the largest free-living feline species in Europe. In Sweden, the population (2025) is estimated at approximately 1400 individuals and the species is present in most parts of the country although most prevalent in central and northern parts (https://www.naturvardsverket.se/data-och-statistik/vilt/lodjur). Swedish lynx are subject to restricted hunting to keep the population at a viable level. During the last two decades, on average 90 animals were hunter harvested annually in licenced hunting. According to Swedish legislation all carcasses of free-ranging lynx found dead or shot by hunters have to be submitted to the Swedish Veterinary Agency (SVA) for post mortem examination in order to monitor health status and the impact of environmental disturbances on the population [7].

Protozoan species in the genus *Sarcocystis* have an obligatory two-host life cycle with herbivores as intermediate hosts and carnivores or omnivores as definitive hosts. The definitive hosts become infected by ingesting tissues of intermediate hosts containing sarcocysts, the encysted stage of the parasite. Intermediate hosts become infected by ingesting an environmentally resistant stage, the sporocyst, excreted in the faeces of the definitive host [3]. Recently, *Sarcocystis* sporocysts were reported in the intestine/faeces of the Eurasian lynx (*L. lynx*) in China [1] and in the Canadian lynx (*L. canadensis*) from the USA [6].

Here we describe the first record of a muscular sarcocyst in a Swedish lynx.

## Case presentation

As part of the continuous survey of all lynx killed or found dead in Sweden, 96 animals were submitted in 1999 to SVA for postmortem investigation. Necropsy and tissue sampling is performed on all lynx submitted to SVA, but histopathology is usually not done unless gross changes are noted. In 1999, a more detailed study on all lynx carcasses included histopathology screening of multiple tissues. The actual case (SVA V393/99) was an adult female lynx with a body weight of 14 kg. The animal, originating from the county of Jämtland in central Sweden, was trapped and euthanised with a shot to the head in March 1999. The carcass was shipped to SVA where it was subjected to a standard necropsy procedure at the pathology department. The lynx was in a generally good bodily condition and the stomach had remains of a capercaillie (*Tetrao urogallus*). Histologically the animal was noticed having secondary findings of multifocal adrenal cortex hyperplasia and spleen atrophy. Additionally, one *Sarcocystis* sarcocyst was found in a haematoxylin and eosin (H&E) stained histological section of an unspecified skeletal muscle. No accompanying cellular reaction was observed. The sarcocyst was removed from the paraffin block and subjected to ultrastructural study by transmission electron microscopy (TEM) and electron micrographs were produced [8]. The following information is based on photographs on file.

**Fig. 1:**
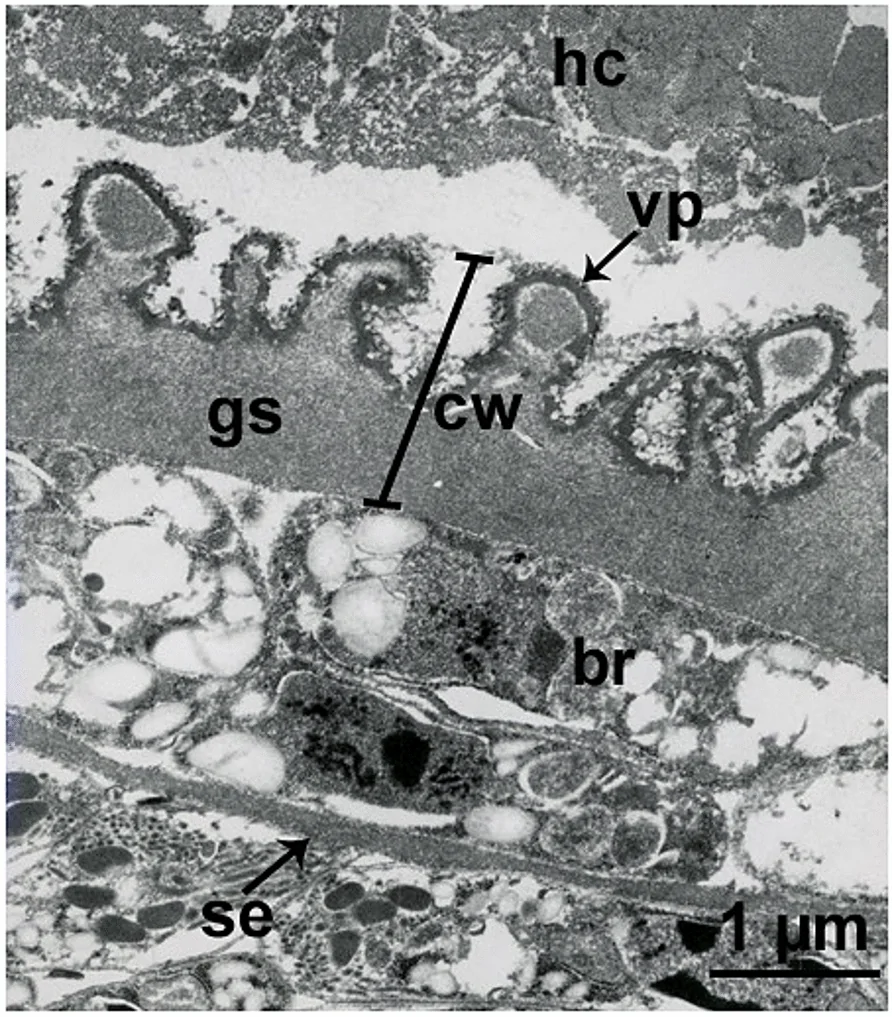
A *Sarcocystis felis*-like sarcocyst in histological section of skeletal muscle of a naturally infected Eurasian lynx (*Lynx lynx*) in Sweden. Note thin sarcocyst wall (opposing arrowheads). H&E

The sarcocyst was cut in cross section while its length remained undetermined. In the H&E sections, the sarcocyst wall was thin (<1 µm thick) with barely perceptible serrations (Fig. 1). The sarcocyst was mature and contained hundreds of bradyzoites.

**Fig. 2:**
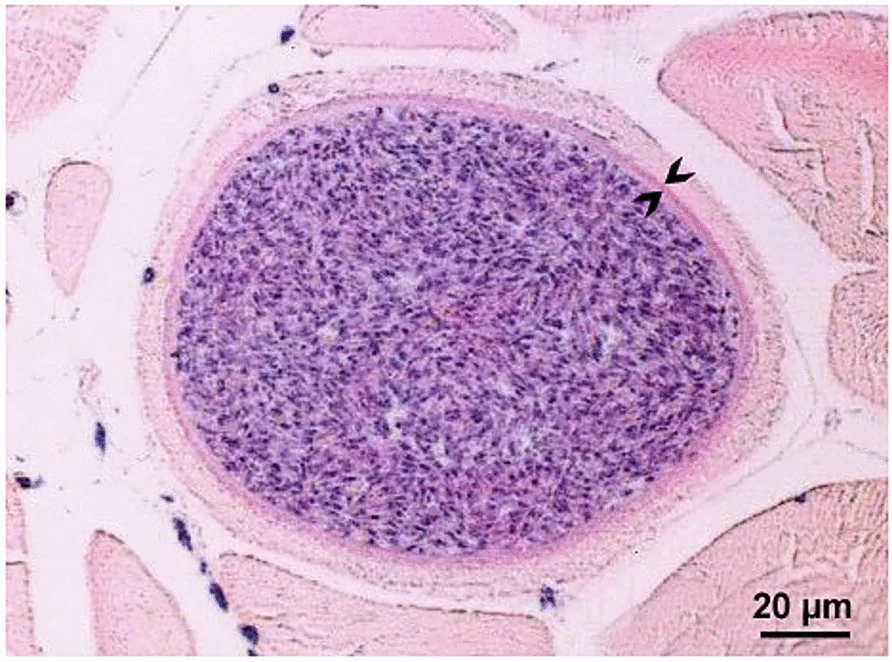
Transmission electron micrograph of a sarcocyst in Fig. 1. Note host cell (hc), villar protrusions (vp), smooth ground substance layer (gs), cyst wall (cw), bradyzoites (br) and septum (se)

Ultrastructurally, the sarcocyst was around 1 µm thick and contained pleomorphic villar protrusions of Type 9c according to the classification of Dubey et al. [3]. The ground substance (gs) was smooth without granules (Fig. 2). Bradyzoites were mature with numerous micronemes, amylopectin granules and up to 3 rhoptries.

## Discussion and conclusions

The present case represents the first published record in a Eurasian lynx (*Lynx lynx*) of a microscopic muscular sarcocyst bearing the morphological features similar to *Sarcocystis felis*, originally described from a bobcat (*Lynx rufus*) from the USA [2]. Until recently all muscular sarcocysts in felids were considered being *S. felis*, but latter studies have shown that there are more than two morphologically distinct sarcocysts in bobcats [4]. As molecular markers are currently lacking for definitive identification of *Sarcocystis* species in felids [5], we designated the parasite in the present report *S. felis*-like. In contrast to the situation with bobcats in the USA where sarcocysts are common [5], it appears that muscular sarcocysts are rare findings in *L. lynx* in Sweden, as based on the limited general histopathology screening that was performed in 1999. Only one single sarcocyst was detected in the actual case, and when an unspecified muscle sample from this lynx was retrieved from the SVA biobank freezer in 2025, no further sarcocysts were found at histopathology even after sectioning through the whole thickness of the muscle sample. In the bobcat the tongue appears to be a prevailing location for *S. felis* [5]. The tongue muscle has not routinely been studied histologically in our material, but it may be a target in future screening to study the prevalence of sarcocysts in lynx in Sweden. Whether the presence of *S. felis* sarcocysts in various feline species is an indication that these are just accidental, aberrant hosts or that they have an as yet undetermined role in the parasite’s life cycle is unknown [5].

## Data Availability

Electron micrographs analysed from the current case are available from the corresponding author on reasonable request.

## Abbreviations

H&E: Haematoxylin and eosin
SLU: Swedish University of Agricultural Sciences
SVA: Swedish Veterinary Agency
